# Risk factors associated with cow mortality following field-based cesarean sections: a study in Minas Gerais, Brazil

**DOI:** 10.29374/2527-2179.bjvm012525

**Published:** 2026-03-10

**Authors:** Gabriel de Freitas Gonçalves, Mariana Assunção de Souza, Rafaella Cristina Caetano, Guilherme Nascimento Cunha

**Affiliations:** 1 Centro Universitário de Patos de Minas, Patos de Minas, MG, Brazil; 2 Programa de Pós-graduação em Ciências Veterinárias. Universidade Federal de Uberlândia, Uberlândia, MG, Brazil; 3 Programa de Pós-graduação em Sanidade Animal e Produção Animal nos Trópicos, Universidade de Uberaba, Uberaba, MG, Brazil; 4 Cirurgia Veterinária, Faculdade de Ciências Agrárias e Veterinárias, de Jaboticabal, Jaboticabal, SP, Brazil

**Keywords:** asepsis, cattle, dystocia, surgery, assepsia, bovinos, distocia, cirurgia

## Abstract

This study aimed to identify risk factors associated with maternal mortality in dairy cows undergoing cesarean section under field conditions in the Alto Paranaíba region, Minas Gerais, Brazil. A retrospective cross-sectional study was conducted using records from 104 cesarean sections performed between August 2022 and April 2025. Preoperative, intraoperative, and postoperative variables were evaluated, including the interval between the onset of parturition and surgery, surgical environment, preparation of surgical materials and the surgical team, duration of the procedure, and postoperative complications. Associations between categorical variables and maternal death were analyzed using odds ratios and Fisher’s exact test. The overall maternal mortality rate was 35.58% (37/104). A parturition-to-surgery interval equal to or greater than eight hours and the concomitant occurrence of postoperative metritis and peritonitis were significantly associated with an increased risk of death. Other variables related to aseptic practices were not statistically significant and were analyzed descriptively. These findings indicate that delayed surgical intervention and concurrent infectious complications are the main risk factors for maternal mortality in dairy cows undergoing cesarean section under field conditions, reinforcing the importance of timely decision-making and strict adherence to aseptic protocols.

## Resumo

Este estudo teve como objetivo identificar fatores de risco associados à mortalidade materna em vacas leiteiras submetidas à cesariana em condições de campo na região do Alto Paranaíba, Minas Gerais, Brasil. Foi realizado um estudo retrospectivo de corte transversal com base em registros de 104 cesarianas realizadas entre agosto de 2022 e abril de 2025. Foram avaliadas variáveis pré-operatórias, intraoperatórias e pós-operatórias, incluindo o intervalo entre o início do parto e a cirurgia, o ambiente cirúrgico, o preparo dos materiais cirúrgicos e da equipe, a duração do procedimento e as complicações pós-operatórias. As associações entre variáveis categóricas e óbito materno foram analisadas por meio de razão de chances (*odds ratio*) e do teste exato de Fisher. A taxa geral de mortalidade materna foi de 35,58% (37/104). Um intervalo parto–cirurgia igual ou superior a oito horas e a ocorrência concomitante de metrite e peritonite no pós-operatório estiveram significativamente associadas a maior risco de morte. As demais variáveis relacionadas às práticas de assepsia não apresentaram significância estatística e foram analisadas de forma descritiva. Esses achados indicam que o atraso na intervenção cirúrgica e a presença simultânea de complicações infecciosas constituem os principais fatores de risco para a mortalidade materna em vacas leiteiras submetidas à cesariana em condições de campo, reforçando a importância da tomada de decisão oportuna e da rigorosa adesão aos protocolos de assepsia.

## Introduction

Normal calving in cattle is a spontaneous physiological phenomenon characterized by the delivery of one or more fully developed calves from the uterus. This process can be divided into three stages: preparation, fetal expulsion, and placental expulsion (Almeida & Resende, 2023).

The first stage is characterized by behavioral changes, such as restlessness and discomfort in the pregnant cow, and involves cervical dilation and relaxation. These processes are regulated by maternal and fetal hormones, including cortisol, relaxin, prostaglandins, and oxytocin, which signal the end of gestation and prepare the body for parturition (Oliveira et al., 2023).

The fetal expulsion stage is initially marked by the release of fetal fluids through the vulva and may last up to four hours in multiparous cows and up to six hours in primiparous animals. During this stage, labor progresses with the presentation of the thoracic limbs, followed by the head, shoulders, trunk, and pelvic limbs of the calf, which should be positioned with the dorsum facing upward (Castro & Silva, 2022). However, deviations from normal fetal presentation or maternal conditions may hinder the progression of parturition, resulting in dystocia and requiring obstetric intervention to preserve the health of both the cow and the fetus (Toneloto et al., 2022).

Several factors are associated with an increased incidence of dystocia. These factors may be classified as intrinsic or environmental and are often interrelated. Intrinsic factors include the cow’s age, parity, breed, gestation length, body weight, and body condition score. Environmental factors include nutritional management, the occurrence of metabolic or infectious diseases, housing conditions, uterine torsion, and animal welfare (Prince & Grillo, 2024).

In this context, obstetric monitoring is essential. When clinical or pharmacological interventions are not indicated or fail to resolve dystocia, cesarean section becomes the surgical procedure of choice to preserve maternal and fetal viability (Almeida & Resende, 2023).

The prognosis of cows undergoing cesarean section is strongly influenced by the timing of the intervention and adherence to aseptic principles. Early surgical intervention and adequate control of contamination are associated with higher survival rates, whereas delays and failures in aseptic technique may predispose animals to postoperative complications such as metritis and peritonitis (Dhindsa et al., 2019).

Postoperative peritonitis may develop because of bacterial contamination, leakage of uterine contents, suture failure, or rupture of surgical materials. This condition can lead to the formation of abdominal adhesions, septic processes, infertility, and, in severe cases, death (Dhindsa et al., 2019).

Previous studies have reported variable maternal mortality rates following cesarean section in cattle. In Canada, Bouchard et al. (1994) reported a mortality rate of 24% (38/159) in dairy cows undergoing cesarean section in a veterinary hospital setting. In Brazil, Mello (2015) observed a mortality rate of 21% (6/28) in cows subjected to field cesarean sections.

Given the challenges of bovine parturition and the variability in outcomes following cesarean section, the objective of the present study was to identify risk factors for mortality in dairy cows undergoing cesarean section, with emphasis on preoperative, intraoperative, and postoperative factors under field conditions.

## Materials and methods

The present investigation was designed as a retrospective cross-sectional study based on data collected from dairy cows submitted to cesarean section on farms located in the Alto Paranaíba region, Minas Gerais, Brazil. Ethical approval was granted by the Research Ethics Committee (CEP) of the University Center of Patos de Minas (UNIPAM), under approval number 7.081.398, and the project was registered with the Certificate of Presentation for Ethical Consideration (CAAE) under number 81551024.1.0000.5549.

Data collection consisted of the analysis of veterinary medical records related to the preoperative, intraoperative, and postoperative management of cesarean sections performed in dairy cows. The records covered procedures carried out between August 2022 and April 2025 and were obtained through the application of structured questionnaires developed using Google Forms. These questionnaires were sent to ten veterinarians who routinely perform bovine cesarean sections under field conditions in the Alto Paranaíba region.

The sample size was determined by convenience sampling and included records voluntarily provided by participating professionals. This strategy may introduce selection bias and limit the external validity of the findings and is therefore acknowledged as a limitation of the study. Initial contact with participants was established via WhatsApp, followed by an in-person meeting for presentation of the research proposal and signing of the Informed Consent Form.

Veterinarians eligible for inclusion in the study were those with active registration with the Regional Council of Veterinary Medicine, practicing in the Alto Paranaíba region, Minas Gerais, and who voluntarily agreed to participate in the research. Veterinarians who did not consent, failed to complete the questionnaire, or provided incomplete information were excluded.

The study investigated risk factors associated with maternal death in dairy cows undergoing cesarean section. The variables analyzed were categorized as follows: preoperative variables, including the interval between the onset of parturition and the surgical procedure, preparation of surgical materials (sterilized or disinfected instruments), and preparation of the surgical team (use of cap, mask, surgical gown or apron, and type of gloves); intraoperative variables, including the environment in which the surgery was performed and the duration of the procedure; and postoperative variables, including the occurrence of complications such as metritis and peritonitis and the administration of antibiotic therapy.

To describe the frequency of the studied variables, the proportion of maternal deaths relative to the total number of cesarean sections performed was calculated, and results were expressed as absolute (n) and relative (%) values. Associations between categorical variables and maternal death were evaluated using the odds ratio (OR) with corresponding 95% confidence intervals (95% CI). Statistical significance was assessed using Fisher’s exact test, adopting a significance level of 5% (p < .05). All statistical analyses were performed using R statistical software (R Development Core Team, 2025).

Due to the retrospective nature of the study and reliance on self-reported field records, information bias cannot be ruled out.

## Results

The frequency of maternal death in dairy cows submitted to cesarean section was 35.58% (37/104). This value was higher than that reported by Bouchard et al. (1994), who observed a mortality rate of 24% (38/159) in dairy cows undergoing cesarean section in a veterinary hospital setting in Canada, and higher than that reported by Mello (2015), who described a mortality rate of 21% (6/28) in cows subjected to field cesarean sections in Brazil.

A more recent study conducted by Kenyon et al. (2025) at The Ohio State University Veterinary Medical Center reported a maternal mortality rate of 16.7% (7/42), which is considerably lower than that observed in the present investigation. This difference may be associated with the controlled hospital environment, where standardized aseptic protocols, adequate infrastructure, and intensive postoperative monitoring are available.

Among the 104 cesarean sections evaluated, the most frequent postoperative outcome was the concomitant occurrence of metritis and peritonitis, accounting for 38.46% (40/104) of cases. The remaining postoperative outcomes and their respective frequencies are presented in Table 1.

**Table 1:** Absolute (n) and relative (%) frequency of maternal deaths in cows undergoing cesarean sections according to postoperative outcome, Patos de Minas, Minas Gerais, Brazil, 2025.

**Postoperative outcome**	**Cesarean sections**		**Maternal deaths**	
	**Absolute (n)**	**Relative (%)**	**Absolute (n)**	**Relative (%)**
Metritis + Peritonitis	40	38.46	30	75.00
Metritis	26	25.00	1	3.85
Peritonitis	8	7.69	4	50.00
Suture dehiscence	2	1.92	0	0.00
Culled cow^*^	1	0.96	1	100.00
Cervical prolapse	1	0.96	0	0.00
No clinical alterations	26	25.00	1	3.85
**Total**	**104**	**100.00**	**37**	**35.58**

* Cause of culling was not described. Source: Authors’ data.

Among the evaluated postoperative outcomes, the highest maternal mortality rate was observed in cows that developed concomitant metritis and peritonitis, reaching 75% (30/40). In contrast, cows that did not present this association of infections showed a considerably lower mortality rate, totaling 10.94% (7/64).

Regarding the analysis of risk factors, only two variables showed a statistically significant association with maternal death. The presence of concomitant postoperative metritis and peritonitis was strongly associated with mortality, with an odds ratio (OR) of 24.64 (95% CI: 9.13–66.51; p < .0001). In addition, a parturition-to-surgery interval equal to or greater than eight hours was associated with an increased risk of death, with an OR of 2.62 (95% CI: 1.11–6.19; p = .028), as shown in Table 2.

**Table 2:** Absolute (n) and relative (%) frequencies of statistically significant risk factors for maternal mortality in dairy cows undergoing cesarean sections and their respective odds ratios (OR), Patos de Minas, Minas Gerais, Brazil, 2025.

**Variables**	**Cesarean sections**			OR^*^	**95% CI**	**p value**
	**Total**	**Deaths (n)**	**Deaths (%)**			
Postoperative complications (metritis + peritonitis)	40	30	75.00	24.64	9.13–66.51	< .0001
Parturition-to-surgery interval (≥ 8 h)	61	27	44.26	2.62	1.11–6.19	.028

* OR = odds ratio; 95% CI = 95% confidence interval. p values < .05 were considered statistically significant. Source: Authors’ data.

Other evaluated variables related to the surgical environment, preparation of surgical materials and the surgical team, duration of the procedure, and postoperative antibiotic therapy did not show a statistically significant association with maternal mortality (p > .05). These variables are presented descriptively in Table 3.

**Table 3:** Absolute (n) and relative (%) frequencies of nonsignificant risk factors associated with maternal mortality in dairy cows undergoing cesarean sections, Patos de Minas, Minas Gerais, Brazil, 2025.

**Analyzed factor**	**Category**	**Cesarean sections**	**Deaths**	
			**(n)**	**(%)**
Surgical environment	Corral	73	28	38.36
	Pasture	31	9	29.03
Surgical material preparation	Disinfected	101	37	36.63
	Sterilized	3	0	0.00
Use of cap and mask	No	101	37	36.63
	Yes	3	0	0.00
Use of surgical gown/apron	No	104	37	35.58
	Yes	0	0	0.00
Type of gloves used	Rectal palpation + non-sterile procedure	101	37	36.63
	Rectal palpation + sterile surgical	3	0	0.00
Duration of surgical procedure	≥3 h	10	6	60.00
	< 3 h	94	31	32.98
Postoperative antibiotic therapy	No	2	2	100.00
	Yes	102	35	34.31

Source: Authors’ data.

## Discussion

The maternal mortality rate observed in this study was 35.58% (37/104), which is higher than those reported in studies conducted in hospital settings, reflecting the challenges associated with performing cesarean sections under field conditions. In a hospital-based study carried out in Canada, Bouchard et al. (1994) reported a maternal mortality rate of 24.00% (38/159), whereas Kenyon et al. (2025) observed an even lower rate of 16.70% (7/42) in a university veterinary hospital.

In contrast, when evaluating cesarean sections performed under field conditions in Brazil, Mello (2015) reported a mortality rate of 21.00% (6/28), which is closer to that observed in the present analysis. Taken together, these findings suggest that the results reported may more closely reflect the realities of field-based cesarean sections, although the present study was not designed to directly compare hospital and field environments.

Differences between hospital and field surgical environments have been widely discussed in the literature as determinants of surgical outcomes. As highlighted by Newman (2008), bovine cesarean sections performed under field conditions present important limitations related to aseptic control, availability of adequate infrastructure, and perioperative support when compared with hospital settings. Marchionatti et al. (2024) emphasize that appropriate preoperative skin asepsis is a critical factor in reducing microbial load and preventing postoperative complications, a measure that is often compromised in procedures carried out outside hospital environments. Furthermore, Queiroz et al. (2024) report a higher occurrence of complications, such as infections and postoperative peritonitis, in field-performed cesarean sections, which are closely associated with logistical and technical constraints. Similarly, Vitor (2024) note that the absence of specialized teams and adequate intraoperative monitoring may negatively affect maternal outcomes, reinforcing the structural and operational differences between these surgical contexts.

Regarding the surgical environment, a higher frequency of maternal death was observed in procedures performed in corrals (38.36%; 28/73) compared with those performed in pasture settings (29.03%; 9/31). Although this difference did not reach statistical significance, Bucataru et al. (2023) reported that environments with higher organic load, dust, and fecal contamination may compromise the maintenance of adequate aseptic conditions during surgical procedures. Additionally, Nickodem et al. (2023) reported a high prevalence of enteric pathogens, such as Salmonella spp., in corral environments, providing biological plausibility for the trend observed here but not allowing causal inference.

Among all variables evaluated, only two showed a statistically significant association with maternal mortality: the concomitant occurrence of postoperative metritis and peritonitis, and a parturition-to-surgery interval of eight hours or more. Concomitant metritis and peritonitis were observed in 38.46% (40/104) of the evaluated cows and were associated with a mortality rate of 75.00% (30/40), whereas cows without this association presented a mortality rate of 10.94% (7/64). These findings are consistent with those reported by Silva et al. (2006), Dhindsa et al. (2019), and Várhidi et al. (2024), who identified uterine and peritoneal infections as major determinants of poor prognosis following bovine cesarean section.

With respect to the interval between the onset of parturition and surgical intervention, cows undergoing cesarean section after a delay of eight hours or more exhibited a maternal mortality rate of 44.26% (27/61), whereas those operated on earlier showed a rate of 23.26% (10/43). This finding is consistent with the observations of Campos and Santos (2021), who suggested that prolonged labor contributes to maternal exhaustion, increased tissue trauma, and greater exposure of the reproductive tract to environmental contaminants. Similarly, Giles et al. (2025) emphasized that earlier surgical intervention is associated with improved maternal outcomes.

The remaining evaluated variables did not show statistically significant associations with maternal mortality and should therefore be interpreted as exploratory findings. With respect to the preparation of surgical materials, no maternal deaths were observed in cases in which sterilized instruments were used, whereas higher mortality rates were observed when only disinfection was employed. Although this difference did not reach statistical significance, it is important to note that the lack of statistical association may be related to the fact that most of the evaluated procedures were not performed in accordance with recommended standards for proper aseptic technique, compared with the small number of surgeries conducted in compliance with technical guidelines (Table 3). These guidelines, as described by Hendrickson (2023) and the World Health Organization (2016), emphasize sterilization as a fundamental requirement for surgical safety.

Similarly, low adherence to personal protective equipment was not statistically significantly associated with maternal mortality. Nevertheless, the absence of maternal deaths in the few cases in which sterile gloves were used is consistent with findings reported by Christmann (2020) and Marchionatti et al. (2024).

Prolonged surgical duration was also not statistically significantly associated with maternal mortality. However, procedures lasting three hours or more showed a higher frequency of maternal death compared with shorter procedures, a trend consistent with reports by Eugster et al. (2004) and Spadari et al. (2023).

Finally, although postoperative antibiotic therapy was widely employed, maternal mortality remained high, indicating that the isolated use of antimicrobials is insufficient to prevent unfavorable outcomes. Moreover, the indiscriminate use of antibiotics to compensate for intraoperative failures should be avoided, as this practice may contribute to antimicrobial resistance. In this context, antimicrobials should be used judiciously and rationally as an adjunct to appropriate aseptic practices and sound surgical techniques, as suggested by Oliver et al. (2011) and Marques et al. (2023).

Overall, the findings of this study indicate that maternal mortality following cesarean section in dairy cows under field conditions is primarily associated with delayed surgical intervention and with the concomitant occurrence of postoperative metritis and peritonitis. The remaining evaluated variables, although clinically relevant in surgical practice, did not demonstrate statistically significant associations. Nevertheless, the absence of statistical differences should be interpreted with caution, particularly given the limited adherence to recommended surgical standards observed in most procedures, as discussed previously.

## Conclusion

The present study identified a high maternal mortality rate in dairy cows undergoing cesarean section under field conditions, reaching 35.58% (37/104). A parturition-to-surgery interval of eight hours or longer and the concomitant occurrence of postoperative metritis and peritonitis emerged as the primary risk factors associated with maternal death. Together, these findings emphasize the importance of prompt obstetrical decision-making and reinforce the central role of appropriate aseptic practices in minimizing postoperative complications and improving survival outcomes in dairy cows subjected to cesarean section under field conditions.
